# A depth-first search algorithm to compute elementary flux modes by linear programming

**DOI:** 10.1186/s12918-014-0094-2

**Published:** 2014-07-30

**Authors:** Lake-Ee Quek, Lars K Nielsen

**Affiliations:** 1 Australian Institute for Bioengineering and Nanotechnology, The University of Queensland, Queensland 4072, Australia

**Keywords:** Elementary mode, Extreme pathway, Linear programming, Rank, Parallel, Memory

## Abstract

**Background:**

The decomposition of complex metabolic networks into elementary flux modes (EFMs) provides a useful framework for exploring reaction interactions systematically. Generating a complete set of EFMs for large-scale models, however, is near impossible. Even for moderately-sized models (<400 reactions), existing approaches based on the Double Description method must iterate through a large number of combinatorial candidates, thus imposing an immense processor and memory demand.

**Results:**

Based on an alternative elementarity test, we developed a depth-first search algorithm using linear programming (LP) to enumerate EFMs in an exhaustive fashion. Constraints can be introduced to directly generate a subset of EFMs satisfying the set of constraints. The depth-first search algorithm has a constant memory overhead. Using flux constraints, a large LP problem can be massively divided and parallelized into independent sub-jobs for deployment into computing clusters. Since the sub-jobs do not overlap, the approach scales to utilize all available computing nodes with minimal coordination overhead or memory limitations.

**Conclusions:**

The speed of the algorithm was comparable to *efmtool*, a mainstream Double Description method, when enumerating all EFMs; the attrition power gained from performing flux feasibility tests offsets the increased computational demand of running an LP solver. Unlike the Double Description method, the algorithm enables accelerated enumeration of all EFMs satisfying a set of constraints.

## Background

The elementary flux mode (EFM) framework is an approach to express reaction pathways contained in metabolic networks [1]. An EFM is a minimal set of reactions carrying non-zero fluxes in the correct direction at steady-state. EFMs provide a systematic and rigorous platform to evaluate functional structures contained in metabolic networks and their link to metabolic phenotypes [2]. EFMs are also used in metabolic engineering to improve product yield [3],[4]. The definition and applications of EFMs have been extensively reviewed elsewhere [2],[5]. Most computational approaches formally generate extreme currents due to the common practice of treating reversible reactions as two separate irreversible reactions [6],[7]. For convenience, “EFM” will be used here to refer to both extreme currents and elementary flux modes.

Current computation frameworks for enumerating EFMs are variants of the Double Description method [1],[8]–[10]. Generating EFMs is a hard task [11], and for any large metabolic network requires very (to impossibly) large processor and/or memory capacities. While complete sets of EFMs have been generated for central carbon metabolism, this is not the case for larger metabolic models. High performance computing clusters become the only viable platform for generating EFMs. The deployment on computing clusters is made possible through several recent advances in EFM computation, namely the *combinatorial parallelization* and *divide-and-conquer* approach [12], and the *bit pattern trees* and *born/die* approach [7],[13]. These approaches are based on the classical *Nullspace* approach [9],[14]. Recent elegant strategies for problem sub-division [15],[16] have lessened the physical memory load from an explosion of intermediate flux modes. Load balancing across and the coordination of computing nodes remain to be significant issues, which limit scalability by problem sub-division.

It has been suggested that only a small subset of EFMs carry significant physiologically fluxes, and it would be more sensible to focus on these [17]. Often we are only interested in enumerating a set of EFMs that satisfies a particular set of criteria, e.g., maximum yield of a product and/or biomass. Using conventional algorithms, all EFMs must be enumerated before selecting those that satisfy the criteria. Alternative EFM generation algorithms based on Linear Programming (LP) can incorporate flux criteria directly during EFM generation [18]–[21]. EFMs are generated by constraining reactions to zero flux; *EFMEvolver* utilizes a genetic algorithm [18], while the *k-shortest EFM* utilizes a binary solver [21] for this purpose. Current LP-based algorithms can produce a relatively small sample of EFMs satisfying certain flux criteria but would struggle to generate the complete set. Identifying new EFMs becomes increasingly difficult as every solution is appended to an ever-growing constraint matrix to avoid repeated outcomes.

This paper describes the generation of EFMs using a depth-first search strategy, which is exhaustive, has a constant and low memory load, and can be massively parallelized into independent sub-problems to take full advantage of loosely coupled grid computing infrastructures. With the depth-first search strategy, the enumeration of EFMs becomes a CPU-limited problem. Five sub-models of increasing size (260 to 379 reactions) extracted from the *E. coli* genome-scale model iAF1260 [22] were used for benchmarking against a Double Description method, *efmtool* (Elementary Flux Mode tool, v4.7.1) [7]. The algorithm employs an LP-based termination criterion for branch searching and is the first algorithm that is capable of determining the complete subset of EFMs satisfying a set of criteria without first enumerating all EFMs.

## Methods

### An alternative elementarity test

Consider a reaction network represented by a stoichiometric matrix *S* with *M* metabolites and *R* irreversible reactions, and a flux vector *v* satisfying the pseudo-steady state condition *S* ⋅ *v* = 0, *v* ≥ 0. The flux vector can be expressed using the null space matrix *NS* of *S* as *v* = *NS* ⋅ *t*, where the length of the coefficient vector *t* is equal to the rank of *NS* and to the nullity *DoF* (degrees-of-freedom) of *S*[23]. Any set of *DoF* independent fluxes will fix the flux vector and the individual members of such a set are called free fluxes [24].

Elementarity is normally tested using either the adjacency [25] or the rank test [26]. Both consider the set of active fluxes, $\bar{Z}\left(v\right)=\left\{i:v_{i}\neq 0,1\leq i\leq R\right\}$. For example, the rank test states that the flux vector *v* : *Sv* = 0, *v* ≥ 0 is elementary, if

(1) $$\mathrm{nullity}\left(S_{*,\bar{Z}\left(v\right)}\right)=\left|\bar{Z}\left(v\right)\right|-\mathrm{rank}\left(S_{*,\bar{Z}\left(v\right)}\right)=1$$

Alternatively, we can consider the set of inactive fluxes *Z*(*v*) = {*i* : *v*_*i*_ = 0, 1 ≤ *i* ≤ *R*}. Since the nullity of *S* is *DoF*, it follows from Eq. 1 that an alternative test would be to establish that an elementary flux vector has exactly *DoF–1* inactive free fluxes. This property was demonstrated in the first *Nullspace* approach paper [9]. While this test is not applied in existing EFM algorithms, it does suggest an alternative algorithm to generate EFMs.

### Depth-first search strategy and implementation

**Figure 1:**
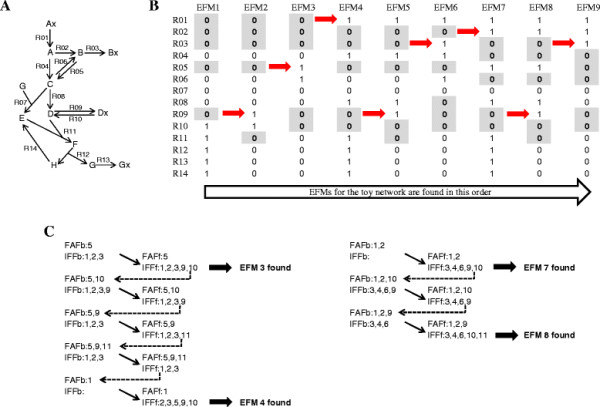
**A worked toy network showing enumeration of EFMs. (A)** Toy network with 14 reactions, 8 internal metabolites, and *DoF* of 6. **(B)** The network has 9 EFMs, sequentially enumerated from left to right by the depth-first search algorithm. Shaded zeros are the leading *DoF-1* IFF for the EFMs. The arrow between a pair of EFM columns (*i* and *i + 1*) denotes the conversion of EFM_*i*_’s most terminal IFF to FAF before EFM_*i+1*_ can be found. The set of IFF is unique for each EFM when only the leading reactions are chosen as pivot rows (see also Additional file 1). **(C)** A list of the sequence of IFF and FAF produced when searching from EFM 3 to EFM 4, and from EFM 7 to EFM 8. Indices of the IFF and FAF are shown. Full arrow indicates forward-tracking; dotted arrow indicates backtracking. Reaction indices in bold indicate successful conversion of a terminal IFF to FAF.

By simple reaction indexing, every EFM in a network can be made to have a unique set of *DoF-1* “**I**nactive **F**ree **F**luxes” (IFF). This is illustrated with a toy network (*R* = 14, *DoF* = 6) in Figure 1, where the IFF set for each EFM is indicated by the shaded cells. A systematic search for EFMs by reaction knock-out only needs to test combinations involving exactly *DoF-1* reactions at a time. While there is a maximum of $C_{\mathrm{DoF}-1}^{R}$ combinations, a depth-first search strategy can exploit the uniqueness property (see Additional file 1), where the next EFM in the search has one or more “**F**ixed to **A**ctive **F**lux” (FAF) from the current EFM’s IFF. This feature is marked by the arrows in Figure 1B. Furthermore, the depth-first search can reuse determined network constraints such as linear dependencies and *v* ≥ 0 to further reduce the workload.

**Figure 2:**
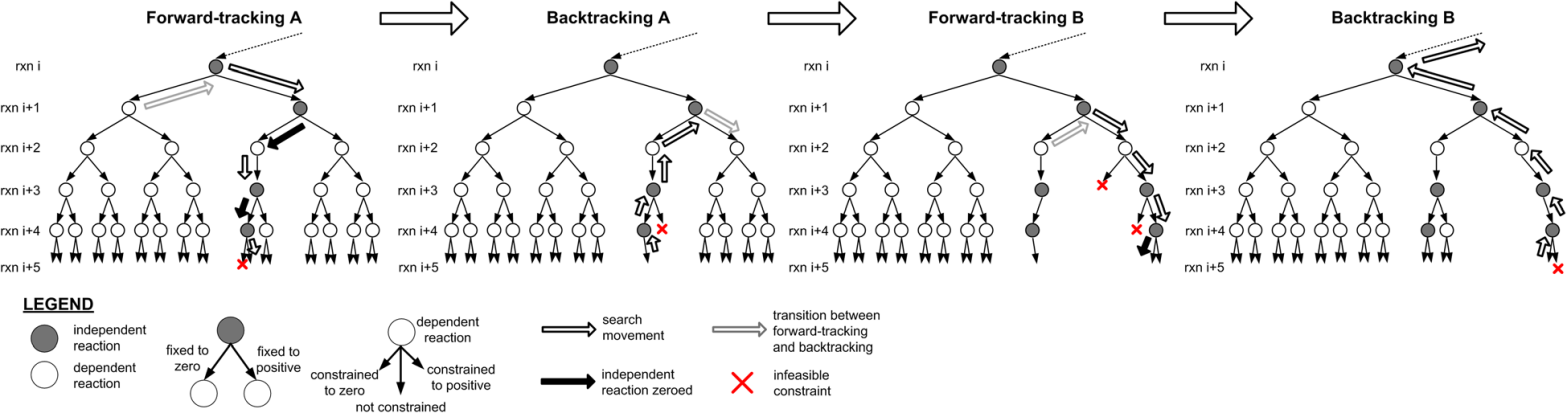
**An illustration of the depth-first search algorithm.** Implementation of the forward-tracking and the backtracking routine for five reactions. Starting from *rxn i*, Forward-tracking A found two (*rxn i + 1* and *i + 3*) downstream IFF. Backtracking A only found *rxn i + 1* being the terminal IFF that can be converted into FAF. Forward-tracking B only found *rxn i + 4* as an IFF. Backtracking B moved upstream beyond *rxn i*, and was unable to find among these reactions any one IFF that can be converted to FAF.

The depth-first search algorithm consists of an alternating pair of forward-tracking and backtracking subroutines (Figure 2). Briefly, forward-tracking finds more downstream IFF (black arrows in Figure 2) by evaluating the feasibility of constraining potential free fluxes to zero. Backtracking finds the terminal (highest index) IFF that can be converted to FAF (the grey arrow in Backtracking A of Figure 2) by testing the feasibility of the new FAF constraint.

**Figure 3:**
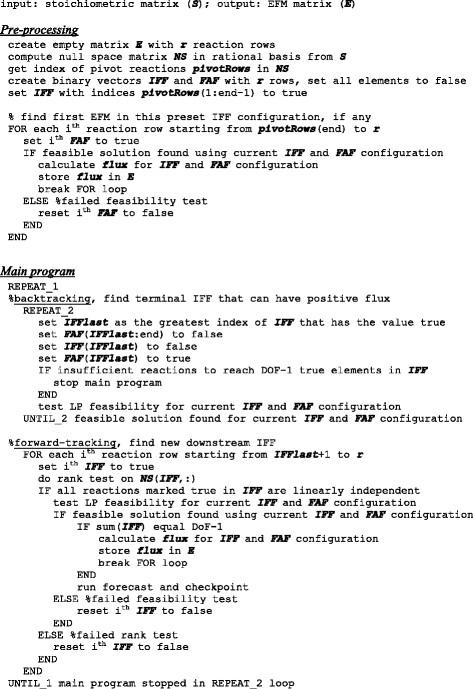
**Pseudocode for the depth-first search algorithm.** Main program consists of an alternating pair of forward-tracking and backtracking subroutines. IFF and FAF are described in pseudocode as binary vectors rather than as index vectors.

The pseudocode for the depth-first search algorithm is provided in Figure 3. During pre-processing, empty FAF and IFF vectors are created, with the IFF vector preset to the leading *DoF-1* pivot rows in a reduced-row echelon form *NS*_*rref*_ of the null space. An EFM may exist for the initial FAF/IFF constraint configuration, which is checked by testing the feasibility of constraining fluxes downstream of the terminal IFF to be a FAF, one reaction at a time.

The main program begins with backtracking. Backtracking consists of a series of feasibility tests that evaluates new constraint configurations where the terminal IFF is sequentially removed from the IFF vector and added to the FAF vector, while all downstream FAF are cleared. The algorithm is switched to forward-tracking if an FAF/IFF constraint configuration is found that produces a feasible solution, otherwise backtracking continues to test the preceding IFF. In forward-tracking, a series of alternating rank and feasibility tests is performed on fluxes downstream of the previous terminal IFF. A candidate flux is added to the IFF vector if it passes both rank and feasibility tests. When the last flux is reached or an EFM is found (i.e., *DoF-1* IFF obtained), the algorithm is switched to backtracking. The search is terminated when there are insufficient downstream fluxes to achieve *DoF-1* IFF.

Two examples of the search sequences are shown in Figure 1C. Between finding EFM_3_ and EFM_4_, four sets of backtracking and forward-tracking were performed. The IFF for EFM_3_ are fluxes 1, 2, 3, 9 and 10. While the conversion of fluxes 10 and 9 into FAF during backtracking each produced a feasible solution, only the conversion of flux 1 into FAF led to EFM_4_.

The feasibility test is achieved by solving an LP problem with a blank objective function

(2) $$\min_{t}\;\bar{0}$$

subjected to:

(3) $$t\geq \bar{0}$$

$NS_{\mathrm{rref}}\cdot t\geq \bar{0}$ for unconstrained fluxes

$NS_{\mathrm{rref}}\cdot t>\bar{0}$ for FAF

$NS_{\mathrm{rref}}\cdot t=\bar{0}$ for IFF

The values in vector *t* are by default zero or greater because *v* ≥ 0 and the coefficients contained in the pivot rows of *NS*_*rref*_ are either zero or one.

### Search speed-up

It is possible to reduce traversal down search trees that cannot yield any EFMs due to insufficient downstream fluxes to form *DoF-1* IFF. Every new IFF introduced may force other fluxes to be active, and flux variability analysis (FVA) can be used to identify these fluxes for the current IFF/FAF constraint configuration. In the toy network (Figure 1), for example, if R01 is an FAF, then R04 must be active when R02 becomes the new IFF.

A recording matrix *M*_*record*_ of *R* rows and *DoF-1* columns is created to register fluxes that are dependently constrained to be active for every new IFF added. Namely, the *j*^th^ column of *M*_*record*_ is a {0, 1} vector, with “1” to mark new reactions that are permanently active as the result of introducing *j*^th^ IFF during forward-tracking. Marked fluxes are no longer candidates for IFF, and are bypassed during forward-tracking. During backtracking, all entries in the *j*^*th*^ column of *M*_*record*_ are reset to 0 when the *j*^*th*^ IFF is converted to FAF.

Progress check-points are implemented to evaluate whether there are sufficient downstream fluxes to reach *DoF-1* IFF. Forward-tracking can only proceed if (1) the number of existing and candidate IFF is equal to or greater than *DoF-1*, (2) there are still *DoF-1* free fluxes among the set of reactions comprised of existing and candidate IFF, and (3) a nullity of zero is obtained for the sub-stoichiometric matrix comprised of columns corresponding to the marked reactions in *M*_*record*_ and the FAF (Additional file 1). For the third check-point, if the nullity is determined to be one, then forward-tracking has found an EFM and can be terminated without finding *DoF-1* IFF. The search algorithm is terminated when backtracking has removed all IFF and forward-tracking has failed at the second check-point (Figure 3).

### Problem parallelization and yield constraints

The flux space can be divided into exclusive subspaces to be separately searched by controlling the participation of all or a subset of basis vectors in *NS*_*rref*_. Each search thread generates EFMs that have a specific configuration of active and inactive fluxes among the pivot reactions. The LP formulation is similar to Eq. 2, except that the parameters contained in the vector *t* are segregated into three types

(4) $$t=\left[\begin{array}{c} t_{\mathrm{free}}\\ t_{\mathrm{inactive}}\\ t_{\mathrm{active}} \end{array}\right]$$

where $t_{\mathrm{free}}\geq \bar{0},\;t_{\mathrm{inactive}}=\bar{0}\;\mathrm{and}\;t_{\mathrm{active}}>\bar{0}$

It is also straightforward to introduce yield or flux constraints into Eq. 2, such as product flux and growth rate for a fixed substrate uptake. The depth-first search would only generate EFMs satisfying these constraints. For example, a flux constraint where the flux of reaction *r* is to be greater or equal to the value *k* can be added to the LP problem as

(5) $$v_{r}=NS_{\mathrm{rref}\;r,*}\cdot t\geq k$$

### Metabolic sub-models

Five metabolic sub-models of increasing size derived from iAF1260 were used to evaluate the computation time required by our approach and *efmtool* to enumerate the full EFM set [7],[22]. The different model sizes were obtained by manipulating the number of biosynthetic outputs from glucose. First, a reduced version of iAF1260 was obtained by extracting, using FVA [27], a subset of reactions (486 out of 2382) that can carry fluxes when biomass yield on glucose is constrained to the maximum. Next, reversible reactions were decomposed into an opposing pair of irreversible reactions and the single biomass equation in the reduced iAF1260 was replaced by 63 individual biomass component drains. The final reduced model has 561 reactions. Five sub models were spawned from this reduced model by increasing the number of allowable biomass drains from 10 to 32 (Table 1). All five models were compressed prior to EFM computation (Additional file 2).

**Table 1:** Properties of the five test models before and after compression

**Model ID**	**Biomass drains (number of drains)**	**Before compression**			**After compression**		
		**rxns.**	**mets.**	**DoF**	**rxns.**	**mets.**	**DoF**
*subaa*	Ala, Asp, Glu, His, Ile, Leu, Lys, Phe, Ser, Val (10)	209	180	57	42	25	23
*aa*	all amino acids (20)	260	221	68	59	30	36
*aarna*	aa and RNA (24)	289	242	73	69	32	41
*aant*	aarna and DNA (28)	312	256	83	74	32	46
*aantpe*	aant and phospholipids (32)	379	317	90	82	35	51
reduced iAF1260	all biomass components (62)	561	454	128	121	41	80

Test models were made incrementally larger by increasing the number of biomass precursors synthesized. A reduced iAF1260 model generating all biomass components is provided as reference. See Additional file 2 for model compression algorithm.

### Hardware and software

The depth-first search algorithm to generate EFMs was tested on The University of Queensland’s Barrine Linux High Performance Computing Cluster. The computer nodes used were equipped with Intel Xeon L5520 dual socket quad core processors (8 × 2.26GHz) and with 24GB memory. Each job was allocated one CPU and 1GB memory.

All algorithms were scripted in MATLAB (R2011a) (The MathWorks Inc., MA), and could be run entirely on a local computer. For parallel computation, the MATLAB scripts were compiled using MATLAB Compiler Runtime (MCR) v7.15 (Linux version) as a standalone application and subsequently deployed on the Barrine cluster. Pre- and post-processing were carried out in a local Linux computer with an Intel Core i7 860 processor (2.93GHz) and 8GB memory. LP optimizations were performed using Cplex Class API provided in IBM ILOG CPLEX Optimization Studio (v12.3) (IBM Corp., NY) for MATLAB, or Gurobi Optimizer (version 5.0). The depth-first search algorithm is provided as Additional file 3.

Computation time was compared against *efmtool*[7]. *efmtool* was implemented within *CellNetAnalyzer* using MATLAB [28]. Since *efmtool* is a multi-threaded program, an entire Barrine node (8 2.26GHz CPUs, 24GB memory, 20GB maximum JAVA heap memory) was allocated to *efmtool* for each run.

The performance of *efmtool* and the depth-first search strategy was benchmarked using the cumulative CPU time (tCPU) obtained using Linux’s *top* command, which is equivalent to real-world time for a processor core working at 100% capacity. For the depth-first search algorithm, the deployed sub-jobs consistently ran at 100% processor capacity, and since only a single processor was allocated to each sub-job, the real-world time recorded in the MATLAB environment is the same as tCPU. This was manually verified. Only the time spent searching the modes is recorded, i.e., pre- and post-processing time was excluded, and the tCPU reported for a given sub-model (in Table 2) is the sum of computation times for the sub-jobs.

**Table 2:** **Time comparison between the depth-first search algorithm and** ***efmtool***

**Model ID**	**No. EFMs**	**tCPU (s)**			**Time ratio**	
		**CPLEX**	**Gurobi**	***efmtool***	**CPLEX /*efmtool***	**Gurobi /*efmtool***
*subaa*	1,779	357	29	10	36	3
*aa*	70,743	54,824	5,198	47	1166	111
*aarna*	126,831	824,230	78,394	38,551	21	2
*aant*	282,027	3,504,514	346,564	731,824	5	0.47
*aantpe*	2,712,435	24,297,990	2,759,912	>6,929,770	< 4	<0.40

Both CPLEX or Gurobi solvers were tested. Absolute time and time ratios with respect to efmtool are shown. For model *aantpe*, *efmtool* failed at the 27th of the 39th iteration.

*efmtool* variably uses a few to all processor cores. In order to exclude pre- and post-processing computation time, the iteration “window” was identified using %CPU given by the *top* command (Additional file 4). The *efmtool* tCPU observations may be inaccurate for model *subaa* and *aa* because their enumerations were completed within seconds, making the iteration window narrow and difficult to identify. Ideally, tCPU would be the same regardless of the number of processor cores used, but a standard deviation of 10% of the average tCPU was observed when we separately processed the sub-model *aarna* using 3, 4 5, 6, 7 and 8 cores (Additional file 4). This variation, however, is small in comparison to the observed tCPU differences between the depth-first search strategy and *efmtool*.

## Results

### Heuristics to reduce computation time

**Figure 4:**
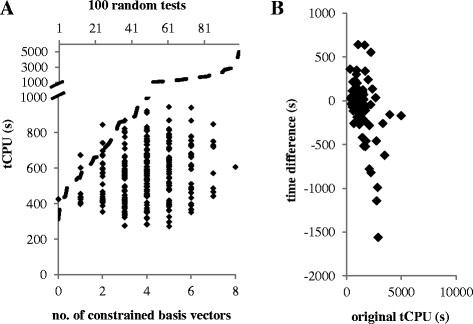
**Total computation time for model*subaa*using different combination (or randomized) of parallelization. (A)** The tCPU for 100 tests using randomized reaction order, sorted in the order of increasing tCPU, are shown with dashed line. The tCPU for all 256 combinations of constraining none, some or all of the 8 reversible basis vectors are shown. The bottom horizontal axis denotes the number of constrained basis vectors in a given combination. **(B)** The differences in tCPU are shown as a result of sorting reaction rows according to the number of possible combination between the positive and negative elements within a row, compared to the original unsorted configuration.

The order in which reactions are processed greatly affects the performance of the Double Description method. A common strategy is to sort reaction rows according to the number of possible combination between the positive and negative elements presented within rows of the null space matrix [16],[29]. In 100 randomly ordered networks based on the (smallest) model *subaa*, computation time using the depth-first algorithm ranged from 310 seconds to almost 5000 seconds (Figure 4A). Row sorting improved computation time for the slowest networks (Figure 4B), whereas several other heuristics failed (Additional file 5).

We also investigated the possibility of using a sparse null space matrix, based on the hypothesis that faster computation can be achieved by minimizing the combinatorial possibilities between the positive and negative elements contained in *NS*. The sparse null space matrix, *NS*_*EFM*_, was made to contain a full independent set of short EFMs (see Additional file 5 for method). Unlike *NS*_*rref*_, *NS*_*EFM*_ is not expressed in a reduced-row echelon form and does not contain *DoF* pivot rows unless the network contains exactly *DoF* EFMs. This means that some EFM basis vectors in *NS*_*EFM*_ can have negative activity coefficients and these basis vectors can be used to parallelize the depth-first search.

The *subaa* model has eight EFM basis vectors that can have negative activity coefficients. We ran all 256 (2^8^) possible combinations of constraining none, some or all of these, and found that tCPU ranged from 270 s to 950 s (Figure 4A). Importantly, the slowest configuration with sparse matrix setup was significantly faster than more than half of the 100 random tests in basic setup. Moreover, parallelization only moderately increased tCPU (Figure 4A); the unparallelized *subaa* model was solved in ~400 s (no basis vector constrained), whereas the fully parallelized model was solved in ~600 s (all 8 basis vectors constrained). The effectiveness of the strategy was confirmed in tests performed on the *aa* model, and was incorporated into the depth-first search algorithm (Additional file 5).

### Performance comparison against *efmtool* using sub-models

The performance of the algorithm was tested using five different metabolic growth models with increasing size derived from a reduced iAF1260 (Table 1). The smallest model *subaa* had 209 reactions and a nullity of 57; the largest model *aantpe* had 379 reactions and a nullity of 90. Network compression removed up to 80% of the original reactions. The observed reduction in the models’ *DoF* was between 43% and 60%. These reductions were attributed primarily to the removal of isolated metabolic pathways (i.e., independent conservation relations), and secondarily to the lumping of duplicated reactions.

The speed of the depth-first search algorithm using Gurobi as the solver was compared against the speed of *efmtool*. For the smaller sub-models *subaa*, *aa*, *aarna*, *efmtool* was found to be faster by 2 to 111 fold, but the depth-first search algorithm was at least 2 fold faster for larger sub-models *aant* and *aantpe* (Table 2). For the largest model *aantpe*, *efmtool* failed at the 27th row iteration (out of 39 steps) due to physical memory limitations, whereas the depth-first search algorithm was able to generate the full set of EFMs within the time it took *efmtool* to fail (31.9 days versus 80.2 days). Gurobi was 10-fold faster than CPLEX, indicating that solver performance is a significant determinant of the computation speed for the depth-first search algorithm. The sets of EFMs generated by either approach were identical in terms of number and form.

**Figure 5:**
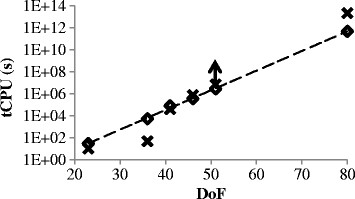
**Comparison of computation times.** Comparing the total computation time (tCPU) in seconds required by the depth-first search algorithm (◊) (using Gurobi solver) and by *efmtool* (×) to generate EFMs for the five test models with increasing size. The tCPU for a model with nullity of 80 was extrapolated based on the trend for the depth-first search.

The depth-first search algorithm, which was executed using MATLAB Compiler Runtime, showed resident memory usages between 94 to 97 MB. Increase in memory usage as individual searches progressed was negligible because solutions were stored in a bit-matrix and written to hard-drive in batches. The computation time increased exponentially with model *DoF* (Figure 5). By interpolation, the search algorithm (using Gurobi solver) would have matched *efmtool*’s speed for a model with a *DoF* of 42. While it was unexpected to find that the test model *aa* showed the poorest time ratio despite it not being the smallest model (Table 2), the trend suggested that the anomaly actually resides in *efmtool*. It is unclear why test model *aa* was more efficiently processed by *efmtool* than other test models, since all models were derived from the same source.

### EFMs for producing valine from glucose

The largest test model *aantpe* was used to demonstrate the potential of generating a complete subset of EFMs satisfying a certain set of flux constraints. Conversion of glucose to valine was used for illustration and, based on the maximum calculated theoretical yield of valine of 0.857 mol per mol glucose, four yield intervals were applied as constraints (Table 3). There is a total of 118,140 EFMs describing the conversion of glucose to valine in the set of EFMs from the full model (Additional file 6). The EFMs and the number of EFMs generated at a given yield interval was found to be the same as the subset extracted from the full EFMs set (using the yield interval as filters). The time required to generate these subsets of EFMs decreased with increasing yield stringency.

**Table 3:** Generating EFMs with different valine yield intervals

**Cutoff**	**Yield interval**	**Number of EFMs**	**tCPU (s)**
99%	≥ 0.85	24	29,195
75%	[0.64, 0.85]	2,472	220,448
50%	[0.43, 0.64]	59,580	2,645,744
25%	[0.22, 0.43]	21,636	5,900,579
0%	≥ 0	2,712,435	24,297,990

EFMs were generated for model *aantpe* using CPLEX solver. The theoretical yields range from 0.004 to 0.8571 mol valine per mol glucose.

## Discussion

The classical Double Description method generates a massive number of intermediate modes before arriving at the final set of EFMs. Existing elementarity tests, the *Adjacency test* and the free-standing rank test [12],[14], provide some attrition power, but neither is sufficient when applied on larger metabolic models. Meeting physical memory requirements to accommodate the combinatorial explosion of intermediate modes is challenging even when using shared or distributed memory system in a computing cluster [7],[12]. Recent elegant strategies for problem sub-division [15],[16] has lessened the physical memory load, though the concomitant increase in computation load [15] limits the potential of how many nodes can be used.

Our depth-first search strategy developed based on an alternative elementarity test: a flux vector is elementary if it has *DoF-1* inactive free fluxes. In fact, all modes surviving the *Adjacency test* in the *Nullspace* approach have this feature [14] (Additional file 1). This EFM feature is the basis for the depth-first search strategy, and crucially mitigates the need for large memory requirements. Unlike existing LP-based approaches, the depth-first search strategy can be exhaustive, since it does not require an ever-expanding constraint matrix. Moreover, the algorithm is readily parallelized using a divide-and-conquer strategy to split the problem into exclusive sub-problems using flux constraints and subsequently enumerate EFMs using as many computing nodes as available with minimal coordination effort. Sub-division of the *subaa* problem into 256 sub-problems, did not significantly increase computation time (Figure 4A). Additionally, the algorithm is robust and running jobs can be interrupted, resumed and/or further sub-divided as desired.

We demonstrated that the LP-based depth-first search strategy is at least comparable in speed to the mainstream *efmtool* (Table 2). Using the Gurobi solver, 86% of the computation time was spent on feasibility testing. The flux constraints *v* ≥ 0, *v* = 0 and *v* > 0, combined with the speed-up strategies, were effective at preventing the search algorithm from traversing down tree branches that did not yield any EFM. Furthermore, finding the next EFM involved only a few enumeration steps because EFM solutions occurred very densely in the search tree. An average yield of 3 solutions per 1000 optimizations was observed. It appears that the attrition power achieved outweigh the additional processor penalty of running an LP optimization compared to approaches based purely on arithmetic operations, particularly for the larger models.

The rank test contributes to a more efficient use of the LP solver. The rank test ensures that flux constraints are only applied to non-redundant reactions, therefore constraints are guaranteed to reduce the flux solution space. Despite the frequency of rank testing, the time spent on calculating matrix rank was only 5% of the total computation time. Matrix rank computation was quick because the matrices evaluated were small—the largest matrix would be a square matrix with the dimension of *DoF-1*—and were largely invariant. In essence, the rank and the LP feasibility tests work synergistically to generate EFMs. In theory, a pure rank or pure feasibility approach could have been used to generate EFMs, but either approach would have required significantly larger number of enumerations than the concurrent use of both.

Our depth-first search approach has not been optimized to reduce computation time. The main component that still requires improvement is the solver performance. The significant speed gained from switching the solver from CPLEX to Gurobi drew our attention to the possibility of stripping down an LP solver to just performing a feasibility test by detecting the presence of an irreducible infeasible set. The optimality criterion is irrelevant here. Additionally, our approach may benefit from more efficient methods to calculate matrix rank [7],[12], rather than MATLAB’s convenient but inefficient *rank* function based singular value decomposition. However, considering the time spent on rank test was only 5% of the total computation time, the benefits would be minor. Lastly, there may be an additional speed gain if our algorithms were to be re-scripted in a native programming language like C++, particularly for large-scale deployment onto computing clusters.

Although this algorithm is suited for large-scale deployment, genome scale enumeration of EFMs in *E. coli* remains impossible with the irreversible version of iAF1260 having a nullity of 985 even after network compression. The smallest, reduced iAF1260 still capable of producing all biomass components has 80 DoF (Table 1) and is projected to require 130 million CPU hours to solve using our setup (Figure 5). The largest number of EFMs enumerated to date is for 227 million for *E. coli* and 2 billion EFMs for *P. triconutum*, respectively [15],[30]. The *P. triconutum* model [15], which has 106 irreversible reactions and a *DoF* of 52 after compression (from 318 reactions). The size of our largest test model (82 reactions, 51 *DoF*) is significantly smaller in comparison to these achieved scales. The preliminary speed results nonetheless demonstrated that the depth-first search strategy is a viable alternative.

Of greater practical importance, this LP approach can generate a complete subset of EFMs satisfying certain flux criteria without enumerating all EFMs first. The potential time saving is very significant as illustrated with the valine example; the time required to generate all EFMs with a yield greater than 75% of the theoretical yield was only 1% of the computation time required to generate a full set of EFMs (Table 3). Constraints derived from thermodynamics and regulation can be incorporated as well [30]–[32]. Instead of a holistic approach, metabolic pathway analysis may benefit from first establishing a narrower, targeted, well-defined metabolic hypothesis, and subsequently generating EFMs that are pertinent to the investigation.

## Conclusions

A depth-first search algorithm to generate EFMs based on linear programming and rank test was developed. The speed is comparable to the conventional approaches based on the Double Description method and the algorithm is scalable, has a low and constant memory load, and can incorporate additional flux constraints to generate a full subset of EFMs of interest.

## Competing interests

The authors declare that there are no competing interests.

## Authors’ contributions

LEQ conceived and designed the method, generated the results and drafted the manuscript. LKN revised the manuscript. Both authors have read the manuscript and approved the final version.

## Additional files

## Supplementary Material

Additional file 1: **A brief example to demonstrate that modes passing the*Adjacency test*always contain*DoF-1*independent zero fluxes.** Also contains demonstration using the toy network that the selection of inactive free fluxes can be made unique if the leading reactions are chosen. Click here for file

Additional file 2: Details on the method used for network compression. Click here for file

Additional file 3: Contains MATLAB scripts for the depth-first search algorithm. Click here for file

Additional file 4: **Contains *efmtool*’s tCPU s for the five sub-models and tCPU test results for running sub-model** ***aarna*** **separately using 3, 4, 5, 6, 7 and 8 cores.** Click here for file

Additional file 5: **Results to show the effectiveness of different heuristics at reducing tCPU.** Details on the method used to generate EFM basis vectors, which are then used to generate all others EFMs. Click here for file

Additional file 6: Graph of EFMs with non-zero valine yield coefficients. Click here for file
