# Supplementary material for: A depth-first search algorithm to compute elementary flux modes by linear programming
Source: BMC Syst Biol. 2014 Jul 30;8:94. doi: 10.1186/s12918-014-0094-2 (PMC4236763; doi:10.1186/s12918-014-0094-2)
Supplement: Additional file 1: — A brief example to demonstrate that modes passing theAdjacency testalways containDoF-1independent zero fluxes. Also contains demonstration using the toy network that the selection of inactive free fluxes can be made unique if the leading reactions are chosen. [file s12918-014-0094-2-S1.docx]

**Unique set of leading *DoF-1* inactive free fluxes**

Here, we will demonstrate that every EFM in a network has a unique set of *DoF-1* inactive free fluxes when reactions are indexed and fluxes with lower indices are preferentially chosen to form the set. In the simplest example, if fluxes *v_1_* and *v_2_* form a dependent set, then *v_1_* will always be chosen as a free flux. More complex relationships arise in larger sets and a general demonstration is provided here.

Let the null space *NS_rref_* be expressed in the reduced-row echelon form

$$NS_{rref}=\left[\begin{matrix} I \\ K \end{matrix}\right]$$

where $I\in\left\{0,1\right\}$ is a *DoF* × *DoF* Identity matrix and $K\in\Re$ is a (*R*-*DoF*) × *DoF* matrix. All basis vectors contained in *NS_rref_* are elementary because the diagonal Identity matrix makes it impossible to produce a simpler vector from any pair of basis vectors by linear combination. Note that the rows of *I* are often referred as pivot rows. Fluxes corresponding to the pivot rows form a set of free fluxes.

Now let the first column of *NS_rref_* be an EFM such that we have

$$\begin{matrix} NS_{rref}=\left[\begin{matrix} 1 & 0 \\ 0 & I' \\ 0 & K_{1} \\ K_{2} & K_{3} \end{matrix}\right] \\ I'\in\left\{0,1\right\},\text{ }K_{1},K_{2},K_{3}\in\Re,\text{ }K_{2}>0 \end{matrix}$$

where $I'$ is a (*DoF-1*) × (*DoF-1*) Identity matrix, and $v'=\left[\begin{matrix} 1 & 0 & 0 & K_{2} \end{matrix}\right]^{T}$is the EFM in question. Fluxes corresponding to rows of $I'$ and $K_{1}$ are candidates that can form the *DoF-1* set of inactive free fluxes for $v'$. Suppose that all reactions in the network have been indexed. The *DoF-1* inactive free fluxes are the reaction rows mapped to$I'$, and $K_{1}$ contains the linear dependent rows with respect to $I'$. A dependent row from $K_{1}$ can be converted into a pivot row by performing Gauss-Jordan elimination on the dependent row, which effectively swap the dependent row with an existing pivot row from $I'$. To reach the described uniqueness, these swaps are performed whenever the index of a dependent row is lower than the index of the pivot row being swapped. Reaction indices mapped to the final $I'$ will always be the same regardless of $I'$starting indices. Thus $v'$ has a unique set of *DoF-1* inactive free fluxes when fluxes with lower indices are preferentially chosen to form the set.

The toy network used in Figure 3 is used to demonstrate that, for every EFM in the network, there exists a unique set of *DoF-1* inactive free fluxes when fluxes with lower indices are preferentially chosen to form the set. The following figure shows that for a given pivot element (marked in yellow, *I**), all corresponding dependent elements (marked in green, *K_1_**) will have greater indices than the pivot’s. For example, in EFM_1_, the pivot element at position [2, 2] corresponds to *v_1_*, and the dependent elements at positions [7, 2] and [10, 2] correspond to *v_4_* and *v_8_* respectively. EFM_7_ has exactly *DoF-1* inactive fluxes and therefore lacks any dependent inactive fluxes. Note that *v_7_* is permanently inactive (i.e., blocked reaction), and can never form a pivot row.

**Placement of Independent zero fluxes**

The placement of the zero flux constraint can be observed in the *Nullspace* approach used in generating EFMs.


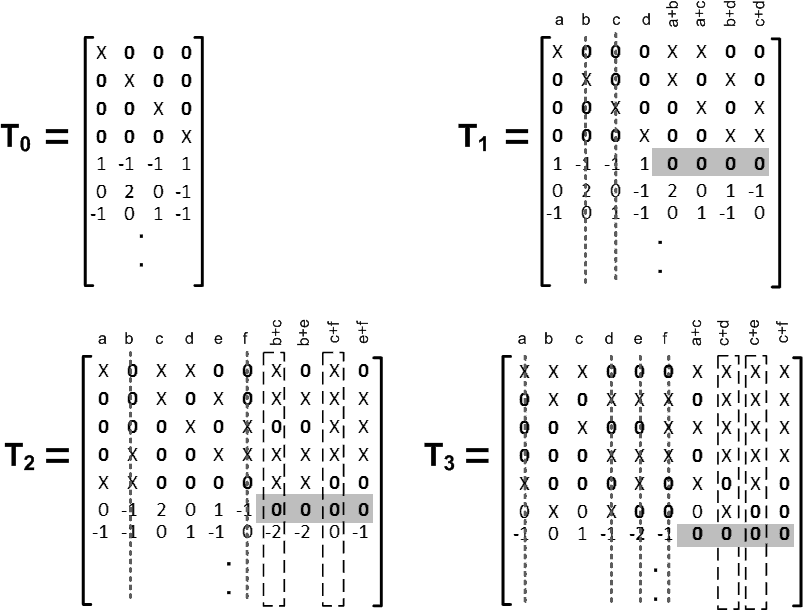


The above is a brief demonstration of the placement of independent zero fluxes in *Nullspace* approach. *T_0_* is the reduced row echelon form of *NS*. In the binary sub-matrix [0,X], the zeros in bold represent the independent reactions being zeroed, which always add up to *DoF-1* (3 in this case). The grey cells containing zero represent the new independent zero flux obtained from flux cancellation by combining two columns. Old columns marked by dashed lines are eliminated because they have negative coefficient in the top row of the real number sub-matrix. New columns produced by combining non-adjacent columns are contained in dashed box, and will not be used in the next round of combination. A child column is elementary only if *DoF-2* independent reactions with zero fluxes are retained after combining the pair of elementary parent columns—another independent zero flux is gained from the flux cancellation. The placement of independent zero fluxes not necessarily occur in the leading *DoF-1* zeros, as shown in column 7 of matrix *T_3_*.

**Depth-first search algorithm in MATLAB code**

input: stoichiometric matrix (*S*); output: EFM matrix (*E*)

*Pre-processing*

E=[]; %empty EFM matrix

NS=**null**(S); %compute null space

[NS_rref_,pivotRows]=**rref**(NS'); NS_rref_=NS_rref_'; [m,n]=**size**(NS);

IZR=**false**(m,1); FPR=**false**(m,1); %binary vectors for IZR and FPR

IZR(pivotRows(1:end-1))=true; %preset IZR to leading DoF-1 pivot rows

%check for possible EFM in this preset IZR configuration, only one if any

**for** i=pivotRows(end):m

FPR(i)=true;

[flux,isFeasible]=**checkByLP**(IZR,FPR,NS_rref_);

**if** isFeasible **storeEFM**(flux); **break**; end

FPR(i)=false;

**end**

*Main program*

**while** true

%backtracking, find terminal IZR that can have positive flux

**while** true

IZRlast=**find**(IZR,1,'last'); %index of terminal IZR

FPR(IZRlast:end)=false; %clear constraints downstream terminal IZR

IZR(IZRlast)=false; FPR(IZRlast)=true; %swap terminal IZR to FPR

%stop algorithm if there never will be sufficient IZR

stopSearch=**checkTermination**(IZR,IZRlast,NS_rref_);

**if** stopSearch **return**; **end**

%if feasible solution found, proceed to forward-tracking

[flux,isFeasible]=**checkByLP**(IZR,FPR,NS_rref_);

**if** isFeasible **break**; **end**

**end**

%forward-tracking, find new downstream IZR

**for** i=IZRlast+1:m

IZR(i)=true;

%do LP if all IZR rows are linearly independent

**if** **rank**(NS_rref_(IZR,:))==**sum**(IZR)

[flux,isFeasible]=**checkByLP**(IZR,FPR,NS_rref_);

**if** isFeasible

**forcast&checkpoint**(i,IZR,FPR,NS_rref_,S);

%if DoF-1 IZR reached, store EFM found & go to backtracking

**if** **sum**(IZR)==n-1 **storeEFM**(flux); **break**; **end**

**continue;** %keep reaction i as new IZR

**end**

**end**

IZR(i)=false;%reaction i has failed rank or LP test

**end**

**end**

*Additional functions*

test new constraint configuration by linear programming

**function** [flux, isFeasible]=**checkByLP**(IZR,FPR,NS_rref_)

[m,n]=**size**(NS_rref_);

%set up LP constraint matrices and empty objective function

A=[NS_rref_;NS_rref_(FPR,:)];

b=[**zeros**(m,1);**ones**(**sum**(FPR,1),1)];

Aeq=NS_rref_(IZR,:); beq=**zeros**(**sum**(IZR),1); f=**zeros**(n,1);

[t,fval,ef]=**linprog**(f,-A,-b,Aeq,beq);

flux=NS_rref_*t;

isFeasible=ef==1;

**end**

check if the whole search can be stopped

**function** stopSearch=**checkTermination**(IZR,IZRlast,NS_rref_)

stopSearch=false;

%IZR must be empty before stopping

**if** **any**(IZR) **return**; **end**

[m,n]=**size**(NS_rref_);

%insufficient downstream reactions or independent reactions

**if** m-IZRlast < n-1 || **rank**(NS_rref_(IZRlast+1:end,:)) < n-1

stopSearch=true;

**end**

**end**

check if forward-tracking can be stopped early

**function forcast&checkpoint**(i,IZR,FPR,NS_rref_,S,flux)

[m,n]=**size**(NS_rref_);

***generate*** *v_pos_* %reactions with permanent positive flux, by FVA

vzeroable_downstream=~v_pos_;

vzeroable_downstream(1:i)=false; %reactions that can be constrained to zero

*allow forward-tracking to continue, if all check-point are satisfied*

1. **sum**(vzeroable_downstream) + **sum**(IZR) >= m-1
2. **rank**(NS_rref_(vzeroable_downstream | IZR,:)) == m-1
3. **size**(S,2) - **rank**(S(:,v_pos_)) == 0

%from condition 3, EFM found even if number of izr did not reach DoF-1

**if size**(S,2) - **rank**(S(:,v_pos_)) == 1

**storeEFM**(flux); EFM found, store EFM

**end**

**end**

*store EFM found during forward-tracking*

**function** **storeEFM**(flux)

*write flux vector into matrix E or hard drive*

**end**
