# Supplementary material for: A depth-first search algorithm to compute elementary flux modes by linear programming
Source: BMC Syst Biol. 2014 Jul 30;8:94. doi: 10.1186/s12918-014-0094-2 (PMC4236763; doi:10.1186/s12918-014-0094-2)
Supplement: Additional file 2: — Details on the method used for network compression. [file s12918-014-0094-2-S2.docx]

# Network compression and decompression

Network compression is a simple but crucial pre-processing step before enumerating EFMs. Our network compression algorithm has three key routines: (1) *match*, (2) *lump* and (3) *eliminate*. These three routines are repeatedly performed in the prescribed order until the size of the stoichiometric matrix remains constant. The *match* routine looks for reaction columns in the stoichiometric matrix that are identical or scaled by a single factor, i.e., reaction “isoenzymes”. Among the matched set of reactions, only one is retained. Each matching event reduces the network’s nullity by one. The *lump* routine looks for metabolites that are solely consumed or produced by one reaction, and then lump that single reaction into all other reactions that have produced or consumed the metabolite in question, respectively. In other words, reactions that have one-to-one, one-to-many or many-to-one mapping relationship based on their metabolite interaction can be systematically lumped. Once a single reaction is lumped into the other(s), the metabolite used in the lumping process is discarded along with the reaction. Lumping does not reduce the network’s nullity, but enriches the final network with independent reactions. The *eliminate* routine simply removes zero columns in the stoichiometric matrix. These zero columns are reactions or pathways that have nullity of one and are completely isolated from the main network, and are often designated “independent conservation relations”.

Network decompression is a straightforward reversal of events performed during network compression. Our approach, however, requires the EFM solutions to be stored in binary form. This allows the recorded compression events to be purely qualitative, with the need to track the scaling factors used in both the “lump” and “match” routines. The bulk of the workload need to generate EFM with real numerical coefficients is therefore delegated to the use of MATLAB’s *rref* (row reduced echelon form) or *svd* (singular value decomposition) functions as per required.
