# Supplementary material for: A depth-first search algorithm to compute elementary flux modes by linear programming
Source: BMC Syst Biol. 2014 Jul 30;8:94. doi: 10.1186/s12918-014-0094-2 (PMC4236763; doi:10.1186/s12918-014-0094-2)
Supplement: Additional file 5: — Results to show the effectiveness of different heuristics at reducing tCPU. Details on the method used to generate EFM basis vectors, which are then used to generate all others EFMs. [file s12918-014-0094-2-S5.docx]

# identifying heuristics to improve computation Speed

We attempted to identify possible correlation of computation speed using 100 samples of randomized reaction order when generating EFMs using the depth-first search algorithm for model *subaa*. This was achieved by visualizing the distribution of tCPUs against various sorting/ranking criteria. tCPU did not correlate with the number of non-zero elements contained in the null space matrix (Figure 1A), nor with the total sum of the possible combinations between the positive and negative elements (Figure 1B). Nor was there a strong correlation between tCPU and the number of feasible subspaces generated by constraining the first 10 basis vectors (maximum number of possible subspaces is 210) (Figure 1C). Additionally, there was no discernible pattern of constraining a subset of free fluxes such that tCPU is reduced (Figure 1D). With row sorting (according to the number of possible combination between the positive and negative elements within a row), only the slowest tests (tCPU >2500s) showed significant improvement in computation time (Figure 1E).

**
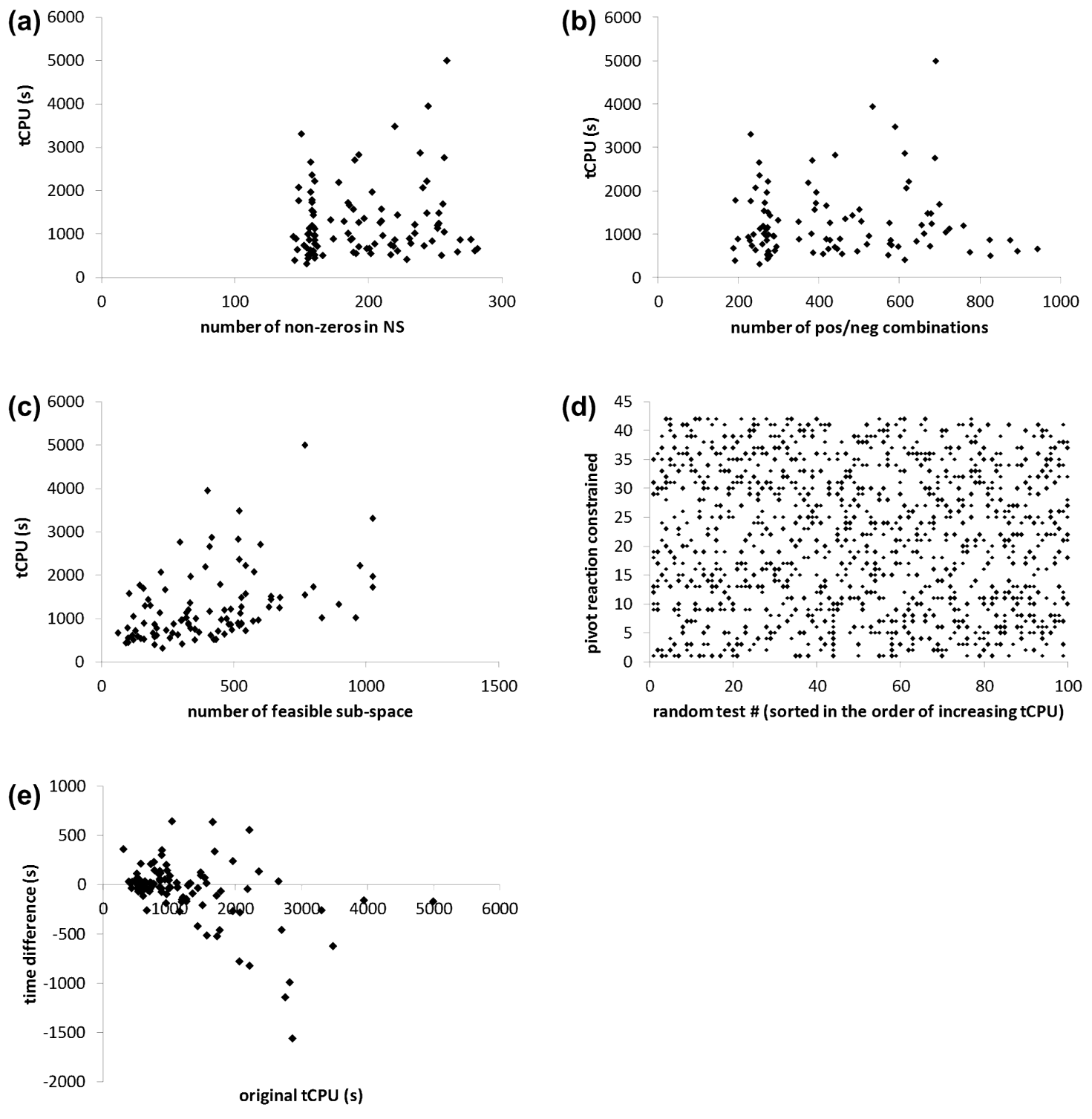
Figure 1.** Computation speed results for 100 sample tests of randomized reaction order.

# Generating a sparse null space matrix

The sparse null space matrix is an independent set of EFMs, with the number of columns equal to the degrees-of-freedom of the stoichiometric matrix. Using a similar approach to generate the shortest EFMs ([de Figueiredo, et al., 2009](#_ENREF_2)), our “greedy” algorithm finds the shortest (and possibly more than one) EFM that involve a given reaction by a mixed integer LP method. To avoid repeated solutions, all previous *N* solutions found are incorporated as constraints at *N+1* solver iteration. Trivial pathway solutions that involve pairing of identical but opposite reactions (*rx* and *ry*) are first identified and then incorporate as constraints as well. The main differences here is that the optimization is performed for a given reaction flux fixed to be positive, whereas in de Figueiredo et al. (2009), the implemented constraint only required at least one reaction having a positive flux to avoid a trivial flux solution.

$$\text{min}\sum_{r=1}^{R} B_{r}$$

$r=1,\text{ }\ldots,\text{ }R \text{ reactions}$*.*

subjected to

$v_{fixed}>0$, $B_{fixed}=1$

$$\text{S}\cdot\bar{\text{v}}=0$$

$$v_{r}\geq0$$

$v_{r}-\omega B_{r}\geq0$, where *ω* is an arbitrary large number

$$B_{soln,n}\cdot B_{r}\leq\left(\sum_{n=1}^{N} B_{soln,n}\right)-1\text{ at the }\left(N+1\right)^{th}\text{ iteration}$$

$$\sum_{r=1}^{R} B_{r}\leq\left\{\begin{matrix} R \text{ at }n=1\text{ iteration} \\ min\left(\sum_{n=1}^{N} B_{soln,n}\right)\text{ at }n>1\text{ iteration} \end{matrix}\right.$$

$B_{rx}+B_{ry}\leq1$, *rx* and *ry* being a pair of identical but opposing reactions.

where

$$v_{r}\in\Re$$

$$B_{r}\in[0,1]$$

$$B_{soln,n}=\left[\begin{matrix} B_{soln,1} \\ \vdots\\ B_{soln,N} \end{matrix}\right] \text{ at }\left(N+1\right)^{th}\text{ iteration}$$

One shortest EFM is generated for each reaction, and these *R* EFMs are compiled into a single matrix. The matrix is filtered for redundant EFMs by trimming the compiled EFMs into an independent set, i.e., the number of remaining EFM columns equals the nullity of the stoichiometric matrix. The trimming process utilizes a rank test. If an EFM column is removed without reducing the rank of the EFM matrix, then it is considered redundant and is therefore discarded. Every column is rank tested until the number of remaining EFMs equals the rank of the EFM matrix, which is in fact *DoF*. A “greedy” approach is employed, whereby longer EFMs are tested first and preferentially discarded.

# parallelization USING independent set of efms

Since a full set of basis vectors spans all flux solutions, it follows that any EFM can be expressed as linear combination of the independent EFMs. Unless the independent set is indeed the complete set of EFM, at least one EFM within this independent set can have negative coefficients, henceforth termed the *reversible basis vector*, in order for the set to generate new EFMs. The flux solution space can therefore be compartmentalized in terms of the participation of these reversible basis vectors, whether they are (a) positive or zero, (b) negative, or (c) unconstrained. These subspaces are defined by the constrained reversible basis vectors and each subspace is distinct from another.

$$v_{EFM,new}=\sum_{i=1}^{I} \alpha_{i}\cdot v_{EFM,i}+\sum_{j=1}^{J} \beta_{j}\cdot v_{EFM,j}+\sum_{k=1}^{K} \gamma_{k}\cdot\delta_{k}\cdot v_{EFM,k}$$

where

$i=1,\ldots,I$ irreversible basis vectors

$j=1,\ldots,J$free reversible basis vectors

$k=1,\ldots,K$ constrained reversible basis vectors

$\alpha_{i},\beta_{j},\gamma_{k}\in\Re$, $\delta_{k}\in\left\{-1,1\right\}$, $\alpha_{i}\geq0$

$$\left(\gamma_{k},\delta_{k}\right)=\left\{\begin{matrix} \left(>0,-1\right)\text{ if the k}^{\text{th}}\text{ vector is constrained to negative} \\ \left(\geq0,1\right)\text{ if the k}^{\text{th}}\text{ vector is not constrained to negative } \end{matrix}\right.\text{ }$$

Some combinations of constrained reversible basis vectors gave lower tCPU(s), while parallelization itself did not increase tCPU(s) significantly (main text Figure 2). More importantly, we observed a repeated subset of constrained reversible basis vectors that were associated with faster computation. The fastest 10 combinations tended to include the reversible basis vectors 2, 3, 7 and 8, while the slowest 10 combinations tended to include the reversible basis vectors 4, 5 and 6 (Table 1). Using the basis vectors’ incidence matrix (Figure 1), we observed basis vectors 2, 3, 7 and 8 have one-to-one mapping to an irreversible basis vector, while basis vectors 4, 5 and 6 do not. This pattern found in the incidence matrix is subsequently used as heuristics to identify good candidates of reversible basis vectors to be constrained such that tCPU is reduced when generating EFMs for the other models.

**Table 1.** Ranking of the fastest and slowest combinations of constrained reversible basis vectors in model *subaa* in the EFM-based parallelization approach.

| Fastest 10 combinations | tCPU(s) | Slowest 10 combinations | tCPU (s) |
| --- | --- | --- | --- |
| 2, 3, 4, 7, 8 | 272 | 1, 3, 4, 5, 6 | 945 |
| 2, 7, 8 | 276 | 1, 2, 3, 4, 5, 6 | 941 |
| 2, 3, 7, 8 | 284 | 2, 3, 4, 5 | 922 |
| 1, 2, 3, 7, 8 | 297 | 1, 4, 5, 6 | 912 |
| 1, 2, 7, 8 | 316 | 1, 2, 4, 5, 6 | 870 |
| 2, 3, 4, 8 | 321 | 1, 4, 5, 6, 7, 8 | 868 |
| 2, 3, 8 | 323 | 4, 5, 6 | 868 |
| 1, 2, 4, 7, 8 | 326 | 1, 3, 4, 5, 6, 7, 8 | 847 |
| 2, 4, 8 | 333 | 5, 6 | 844 |
| 1, 2, 8 | 339 | 1, 3, 5, 6 | 832 |


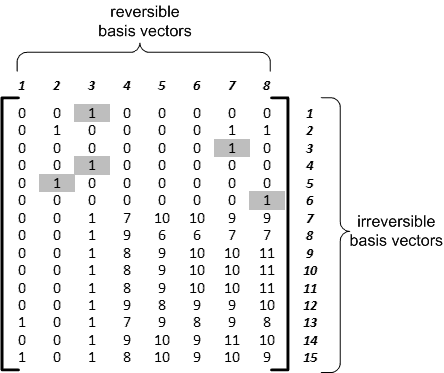


**Figure 2** Incidence matrix of the basis vectors for the test model *subaa*. The incidence matrix shows the number of reactions that are common between two basis vectors. Grey cells highlights reversible basis vectors that have one-to-one mapping to another irreversible basis vector.

The effectiveness of this heuristics was confirmed by time results obtained for a larger model. The incidence matrix for test model *aa* identified 10 basis vectors with a one-to-one mapping to an irreversible basis vector. The tCPU of parallelization constraining these 10 basis vectors was compared to the tCPU of 19 parallelization each using random combinations of constrained reversible basis vectors (Figure 2). Out of the 19 random combinations, only one was found to be faster (by 15%) than the combination prescribed by the heuristics, while the majority was slower (>150% tCPU) (Figure 3).


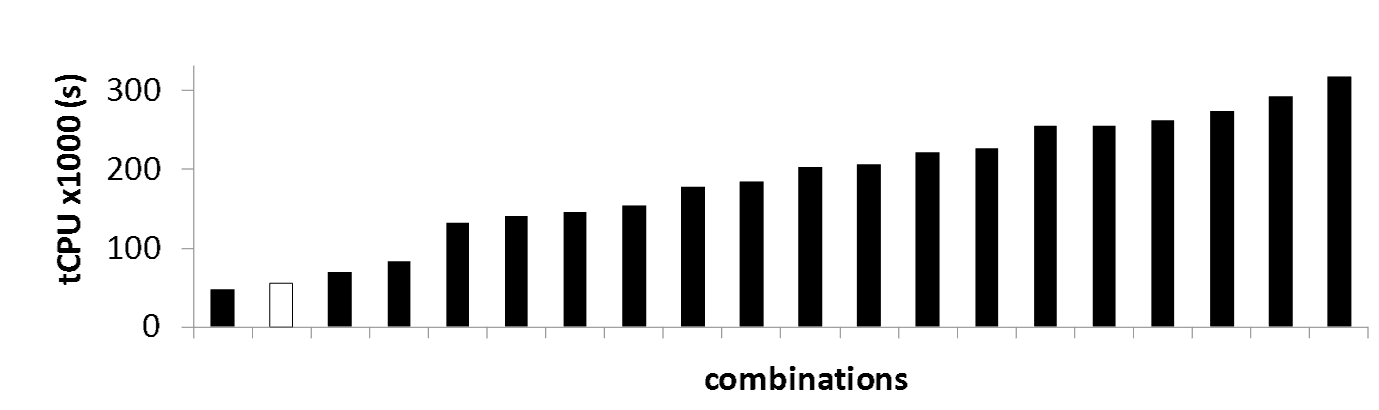


**Figure 3.** tCPUs for test model *aa* when generating EFMs using 20 different combinations of constrained reversible basis vectors. The second fastest combination was chosen using our heuristics, while the remaining 19 combinations were chosen at random.
