# Supplementary material for: A depth-first search algorithm to compute elementary flux modes by linear programming
Source: BMC Syst Biol. 2014 Jul 30;8:94. doi: 10.1186/s12918-014-0094-2 (PMC4236763; doi:10.1186/s12918-014-0094-2)
Supplement: Additional file 6: — Graph of EFMs with non-zero valine yield coefficients. [file s12918-014-0094-2-S6.docx]

**
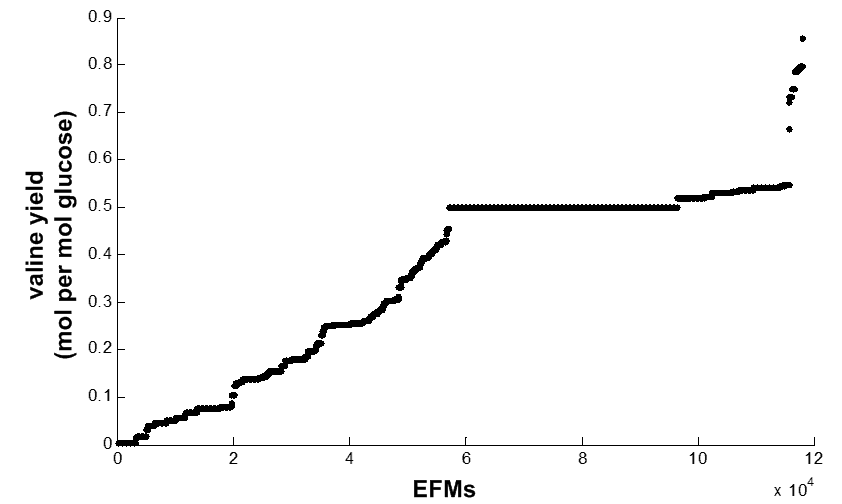
Distribution of valine yields in model *aantpe***

There is a total of 118,140 EFMs describing the conversion of glucose to valine in the set of EFMs from the full model. The theoretical yields range from 0.004 to 0.8571 mol valine per mol glucose. It was interesting to observe that 33% of the valine EFMs have yield at 0.5.
